# Acute Pharmacological Effects of Two Synthetic Cathinones in Humans: An Observational Study of *N*-Ethylhexedrone and *N*-Ethyl-nor-pentedrone

**DOI:** 10.3390/ph18050721

**Published:** 2025-05-14

**Authors:** Melani Núñez-Montero, Clara Pérez-Mañá, Olga Hladun, Lourdes Poyatos, Dolly Andrea Caicedo, Georgina De la Rosa, Martha Catalina Argote, Soraya Martín, Mireia Ventura, Nunzia La Maida, Annagiulia Di Trana, Silvia Graziano, Simona Pichini, Magì Farré, Esther Papaseit

**Affiliations:** 1 National Center on Addiction and Doping, National Institute of Health, Viale Regina Elena 299, 00161 Rome, Italy; nmmelani@gmail.com (M.N.-M.); nunzia.lamaida@iss.it (N.L.M.); annagiulia.ditrana@iss.it (A.D.T.); silvia.graziano@iss.it (S.G.); mfarre.germanstrias@gencat.cat (M.F.); epapaseit.germanstrias@gencat.cat (E.P.); 2 Clinical Pharmacology Department, Hospital Universitari Germans Trias I Pujol (HUGTP-IGTP), Carretera de Canyet s/n, 08916 Badalona, Spain; cperezm.mn.ics@gencat.cat (C.P.-M.); ohladun.germanstrias@gencat.cat (O.H.); lpoyatos@igtp.cat (L.P.); dacaicedo.mn.ics@gencat.cat (D.A.C.); grosalo.germanstrias@gencat.cat (G.D.l.R.); mcargoteo.germanstrias@gencat.cat (M.C.A.); smartins.mn.ics@gencat.cat (S.M.); 3 Department of Pharmacology, Therapeutics and Toxicology, Universitat Autónoma de Barcelona, Cerdanyola del Vallés, 08193 Bellaterra, Spain; 4 Energy Control, Associació Benestar i Desenvolupament, 08041 Barcelona, Spain; mireia@energycontrol.org

**Keywords:** pharmacology, synthetic cathinones, subjective effects, New Psychoactive Substances, *N*-ethylhexedrone, *N*-ethyl-nor-pentedrone

## Abstract

**Background:** Synthetic cathinones (SCs) are the second most representative class of New Psychoactive Substances, with more than 100 analogues identified in the illicit drug market up to 2024. According to the United Nations Office on Drugs and Crimes, *N*-ethylhexedrone (NEH) and *N*-ethyl-nor-pentedrone (NEP) were identified among the most frequently seized SCs worldwide. However, still, little is known with regard to their pharmacological effects in humans. **Methods**: For the first time, we conducted a naturalistic, prospective observational study in 16 participants (7 women and 9 men) with a previous history of psychostimulant recreational use. They intranasally self-administered a single dose of NEP (*n* = 8, 20–40 mg) or NEH (*n* = 8, 20–40 mg). The physiological effects (systolic and diastolic blood pressure, heart rate, and temperature) and subjective effects (visual analogue scales, Addiction Research Center Inventory questionnaire and Evaluation of Subjective Effects of Substances with Abuse Potential questionnaire) were assessed up to 4 h after the self-administration at different time points (0, 20 and 40 min and 1, 1.5, 2, 3 and 4 h). **Results**: Despite several differences, both NEP and NEH produced significant effects within 20 min, with a return to baseline 3–4 h after self-administration. In general, NEP showed a faster onset and a more rapid disappearance of subjective effects than NEH. Moreover, intranasal self-administration of NEH and NEP in experienced recreational drug users, within a non-controlled setting, induces a constellation of psychostimulant-like effects. **Conclusion**: NEH and NEP showed similar pharmacological properties after insufflation, with typical effects of SCs

## 1. Introduction

Synthetic cathinones (SCs) form thesecond most numerous class of New Psychoactive Substances (NPSs), accounting for more than 100 globally identified analogues. Similarly to the other NPS classes, the SCs available on the illicit market vary yearly, reflecting the dynamic nature of the NPS phenomenon [1]. Mephedrone is usually considered the SC lead compound; it was internationally scheduled in 2010 due to the related risks to public health. Afterwards, other analogues, such as methylone, methylenedioxypyrovalerone (MDPV), and α-pyrrolidinovalerophenone (α-PVP), 4-chloromethcathinone (4-CMC) emerged as legal alternatives, presenting similar effects despite the structural differences [2]. One of the most challenging aspects of the NPS phenomenon is the unknown pharmacological and toxicological profile of newly emerging molecules, which complicates the correct diagnosis of intoxicated subjects [3]. Recently, *N*-ethylhexedrone (NEH) and *N*-ethyl-nor-pentedrone (NEP) raised concerns as the latest and most prevalently seized SCs globally [4]. Currently, official epidemiologic data on the NEH and NEP prevalence of consumption are not available. Otherwise, published case reports, toxicology reports and data from drug checking points in sentinel cities confirm the increasing spread of the substances.

First synthesized by Boehringer Ingelheim in 1964, NEH, also known as *N*-ethyl-hex, hex-en, and Hexen, is a synthetic analogue of pentedrone. In 2015, European Monitoring Centre on Drugs and Drug Addiction Early Warning System received the first NEH seizure report, suggesting its trafficking in the illicit market [5]. This substance is typically consumed by nasal insufflation at doses between 30 and 40 mg, with strong effects reported at doses above 50 mg. The limited information on its effects is mostly available on specialized websites and drug fora, whereas scientific data mostly refer to fatal and non-fatal cases [5,6,7]. However, little is known about the pharmacology of NEH [8,9].

Similarly to other SCs, NEH is frequently used in combination with other drugs of abuse or NPSs, comprising other SCs. In pre-clinical studies, NEH was demonstrated to strongly inhibit the dopamine transporter (DAT), norepinephrine transporter (NET), and serotonin transporter (SERT) [10,11,12]. In animal models, data showed that NEH typically induced excitement and stereotypies, and generated place preference behaviour, suggesting a high abuse potential similar to that of other SCs and stimulants [13,14]. Its metabolic profile in urine includes reduction, hydroxylation, *N*-dealkylation, and di-hydrogenation reactions [15]. Recently, documented clinical cases of NEH intoxication alone or in combination with other drugs have been reported [16,17,18,19,20].

NEP, also known as *N*-Ethylpentedrone or Ethyl-pentedrone, is the *N*-ethyl analogue of pentendrone, and *N*-ethylpentylone is its methylenedioxy derivative. These structural modifications increase its potency as a DAT inhibitor by two- to three-fold. Also for this SC, its pharmacological behaviour is primarily inferred from its chemical structure and related animal studies [21]. On Internet fora and specialized websites, recreational users describe short-acting, stimulating, euphoric, and mildly entactogenic effects when consumed by insufflation, with frequent redosing or bingeing. Furthermore, one case of drug-facilitated sexual assault (DFSA) involving NEP has been documented in the literature [22], and some intoxication cases have been reported [17,23,24].

While experimental studies with controlled administration of mephedrone [25,26,27], methylone [28,29,30] and α-PVP [31] have been conducted in humans, data on NEH and NEP toxicity are still limited to preclinical studies, toxicological data in poisonings, surveys and recreational user reports. In the present observational study, we aimed to describe the acute pharmacological effects of human self-administration of NEH and NEP in naturalistic conditions and determine drug concentrations in oral fluid.

## 2. Results

### 2.1. Participants’ Characteristics

In the NEP administration group, participants had a mean age of 31 ± 6 years (range: 24–40 years), weighed 66 ± 12 kg (range: 52–84 kg), and had a mean body mass index (BMI) of 21 ± 2 kg/m^2^ (range: 19–25 kg/m^2^). Four were current tobacco smokers (range: 5–30 cigarettes/day) and all of them except one consumed alcohol (mean: 1.8 standard drink/day). In the NEH administration group, participants had a mean age of 28 ± 5 years (range: 23–39 years), weighed 65 ± 11 kg (range: 43–80 kg), and their mean body mass index (BMI) was 21 ± 2 kg/m^2^ (range: 18–25 kg/m^2^). Only two participants were current tobacco smokers (range: 3–8 cigarettes/day), and all of them, except for two, consumed alcohol (mean: one standard drink/day). All of them were recreational drug users with prior experience with psychostimulants (MDMA, six recreational uses/year; amphetamines, eight recreational uses/year; cocaine, four recreational uses/year) and/or any SCs. Baseline drug urine tests were negative for all participants.

### 2.2. Physiological Effects

**Figure 1:**
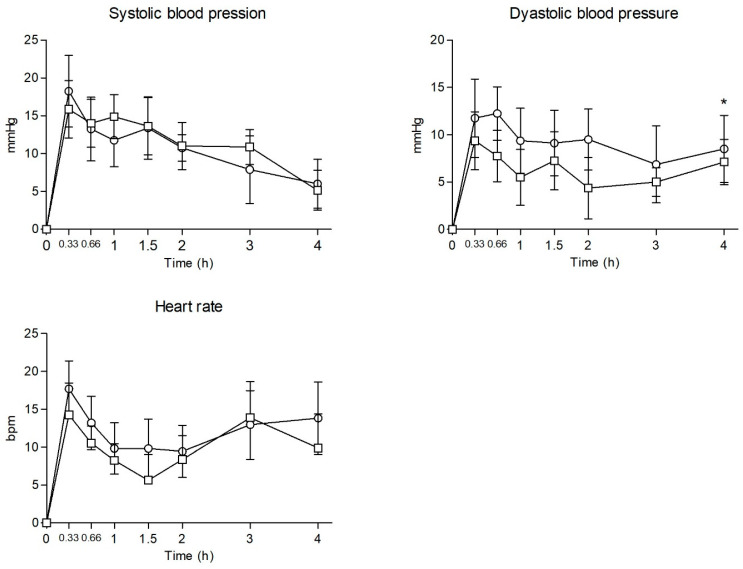
Time courses of systolic blood pressure, diastolic blood pressure, and heart rate after administration of ᵒ NEH (*n* = 8) and □ NEP (*n* = 8). Data points and errors bars represent the mean and standard error of the mean (SEM), respectively. Statistical differences between NEP and NEH are presented as “*”.

The significant statistical findings of NEP and NEH physiological effects are summarized in Table 1, and their time courses are illustrated in Figure 1.

Self-administration of a single dose of NEH and NEP increased systolic blood pressure (SBP), diastolic blood pressure (DBP), heart rate (HR), and temperature (T). For NEH, a similar time course for SBP, DBP and HR was registered, with increases of +22 mmHg, +17 mmHg and +21 bpm, respectively, as the maximum effects (E_max_). Compared to baseline values, statistically significant differences were detected for SBP and DBP from 20 min to 2 h (h). Also, HRs were statistically different from baseline values at 20 min (min) and 3 h.

For NEP, E_max_ values corresponded to a sharp increase for SBP (+21 mmHg) and HR (+22 bpm), while DBP showed a milder increase (+12 mmHg). Compared to baseline values, statistically significant differences were detected for SBP from 20 min to 2 h, for DBP at 20 min, 40 min, 1.5 h, and 4 h, and for HR from 20 min to 4 h.

NEH and NEP produced a minimal rise in cutaneous T (T). Statistically significant differences compared to baseline values were detected only at 4 h for both substances.

Comparing NEH and NEP, E_max_, area under the curve from 0 to 4 h (AUC_0–4h_), or time course (T-C) points presented no statistically significant differences, except for DBP at 4 h.

### 2.3. Subjective Effects

**Figure 2:**
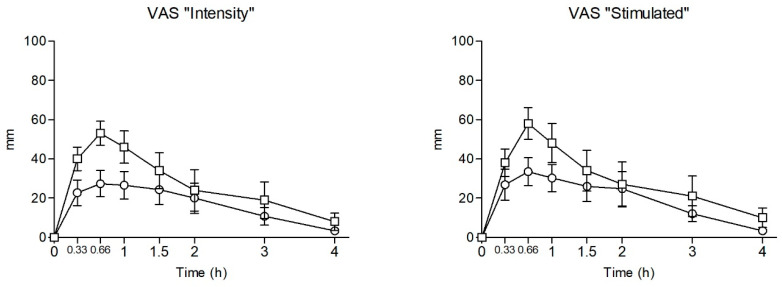

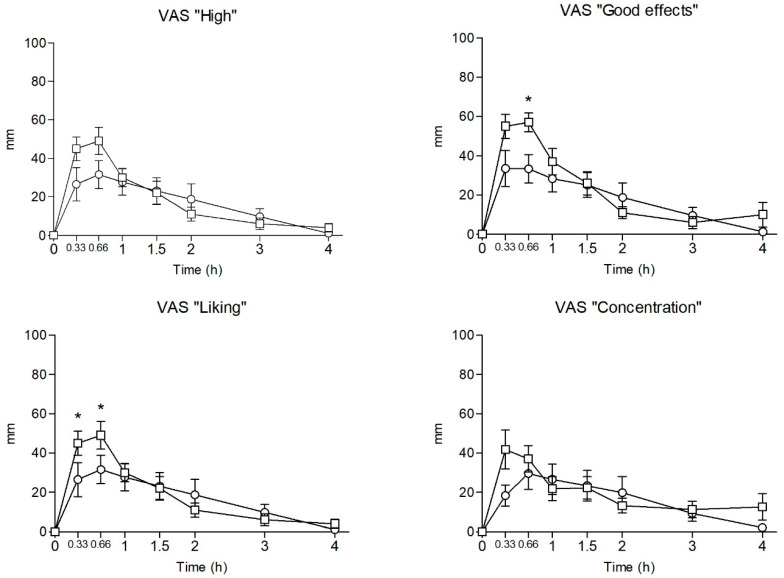
Time course of physiological and subjective effects of NEH and NEP after intranasal self-administration of ᵒ NEH (*n* = 8) and □ NEP (*n* = 8). Data points and errors bars represent mean and standard error of the mean (SEM), respectively. Statistical differences between NEP and NEH are presented as “*”.

Statistical data and T-C points of the observed subjective effects following NEH and NEP intranasal administration are presented in Table 2 and Figure 2, respectively.

Self-administration of a single insufflated dose of NEH and NEP increased VAS (VisualAnalog Scale) scores mainly related to stimulation and well-being (VAS “stimulate”, “high”, “good effects”, “liking”), altered perception (“different body feeling”), and empathy (“open”, “trust”, “feeling close to others”, “I want to hug someone”).

For NEH, the highest E_max_ scores were for the VAS items “feeling close to others” (+43 mm), “open” (+40 mm), “trust” (+39 mm), and “liking” (+39 mm), followed by “stimulated” (+37 mm), “good effects” (+37 mm), “high” (+35 mm), “focused” (+32 mm), “different body feeling” (+28 mm), and “I would like to be with other people” (+28 mm). Compared to baseline values, statistically significant differences were detected for all the mentioned VAS items from 20 min to 2 h, except for “different body feeling”, which showed significant differences at 1 h and 2 h and for “I want to hug someone,. which showed significant differences from 40 min to 1.5 h.

For NEP, the highest E_max_ scores were observed for the VAS items “liking” (+72 mm), “I would like to be with other people” (+68 mm), “open” (+66 mm), “feeling close to others” (+65 mm), and “trust” (+64 mm), followed by “stimulated” (+56 mm), “high” (+51 mm), “focused” (+49 mm), “different body feeling” (+34 mm), and “I want to hug someone” (+34 mm). Compared to baseline values, statistically significant differences were detected for the VAS items “stimulated”, “open”, “trust” and “feeling close to others” from 20 min to 4 h, for “intensity” and “I want to hug someone” from 20 min to 3 h, for “high”, “good effects”, “liking” and “concentration” from 20 min to 1,5 h, and for “different body feeling” and “drowsiness”, at 1 h and 2 h, respectively. After NEP self-administration, the VAS “bad effects” item E_max_ score was measured at +40 mm, with statistically significant differences from baseline detected at 1 h and 1.5 h. Statistically significant differences between NEH and NEP were detected in E_max_ and T-C points for the VAS items “good effect”, “bad effects”, “liking”, “open”, and “I want to be with other persons”. A significant difference was also found in E_max_ for the VAS item “intensity”. For the remaining VAS items, no statistically significant changes were detected after the self-administration of NEH or NEP.

Regarding sexual subjective effects, the E_max_ scores for the VAS item “sexual desire” were +12 mm for NEH and +8 mm for NEP, while the scores for “sexual arousal” were +10 mm for NEH and +4 mm for NEP. No statistically significant differences were detected from the baselines, or between NEH and NEP.

According to the Addiction Research Center Inventory short form (ARCI), NEH produced higher maximum scores compared to baseline on the MBG (euphoria), A (amphetamine effects), and BG (intellectual efficiency) subscales, whereas NEP consumption resulted in higher scores for MBG, A, and PCAG after 2 h. For NEH, statistically significant differences were detected for A and BG from 1 to 3 h, for MBG from 1 to 2 h, and for PCAG at 1 h (lower score). For NEP, statistically significant differences were detected for A from 1 to 4 h, for MBG from 1 to 2 h, and for LSD at 1 h. Differences between NEH and NEP in E_max_, AUC_0–4h_ and T-C points were reported only for PCAG.

Considering the Evaluation of Subjective Effects of Substances with Abuse Potential(VESSPA) questionnaire, significant changes compared to the baseline were detected for the ANX, SOC and PS subscales after NEH self-administration and for the S, ANX, SOC and ACT subscales after NEP self-administration. No statistically significant differences were found for E_max_, AUC_0–4h_, or T-C points between NEH and NEP.

### 2.4. Oral Fluid Concentration of NEP and NEH

**Figure 3:**
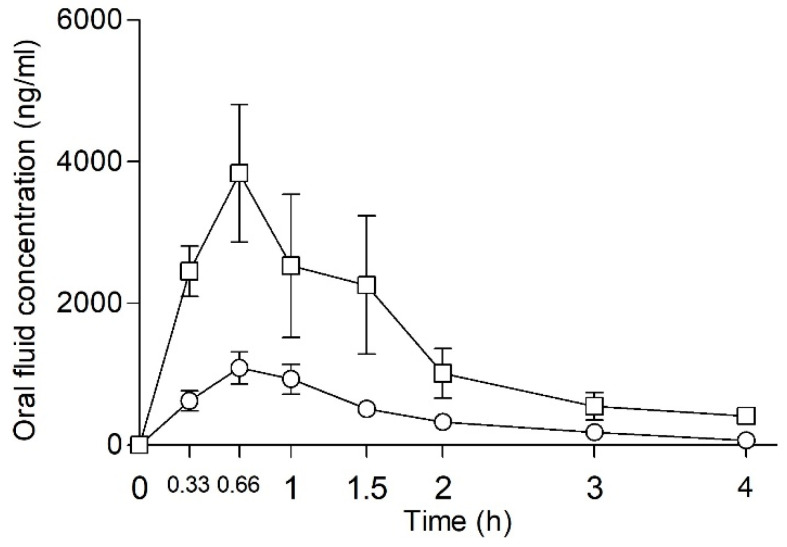
Time course of NEP and NEH oral fluid concentrations after intranasal self-administration (□ NEP *n* = 8, mean dose 33.75 mg; ° NEH, *n* = 7, mean dose 31.43 mg). Data points and errors bars represent mean and standard error of the mean (SEM), respectively.

After intranasal self-administration, NEH concentrations in oral fluid increased rapidly, peaking at 0.83 h after consumption, and decreasing up to 4 h (Figure 3).

To ensure the robustness and reliability of the results, one subject (man, 40 mg) was excluded from the analysis since outlier NEH values were observed, according to the Dixon test. Mean maximum concentration (C_max_) values of 1571 ± 1367 ng/mL (range 18–2999 ng/mL) were obtained at a T_max_ of 2 h after drug administration. The AUC_0–4h_ was 1135 ± 590 ng·h/mL (range 475–1777 ng·h/mL). After 4 h, oral fluid NEH concentrations were detectable only in three out of seven subjects.

Similarly, NEP oral fluid concentrations increased rapidly after intranasal administration, peaking at 40 min, and then rapidly decreasing up to 4 h (Figure 3). C_max_ values of 4950 ± 5545 ng·h/mL (range 1091–14,525 ng·h/mL) were obtained at a T_max_ of 40 min following drug administration. At 4 h, the NEP concentration was 10 times lower in comparison to C_max_. The AUC_0–4h_ was 5689 ± 3815 ng·h/mL (range 1529–13,370 ng·h/mL).

Oral fluid NEH and NEP concentrations exhibited substantial inter-subject variability.

All the administered doses were well tolerated, and no serious adverse events were reported. No local tissue damage to the nostrils or any other potential acute medical complication was reported. Moreover, immediate adverse cognitive effects or alterations in mental state were not observed after the administration.

## 3. Discussion

To the best of our knowledge, this is the first pilot study that describes the acute pharmacological effects in recreational drug users after intranasal administration of NEH and NEP in a single dose, confirmed through the detection of oral fluid concentrations in a non-controlled setting.

The primary finding was that a single intranasal self-administration of both the investigated SCs elicited significant cardiovascular responses and subjective effects, including stimulation, euphoria, well-being feelings, and enhanced social connectedness. Preliminary results suggest that NEP and NEH produce characteristic psychostimulant and connectogenic effects, encompassing empathogenic properties.

Similarly to other amphetamine-like NPSs, NEP and NEH showed the typical excretion profile of nasally administered SCs, exerting significant effects within 20 min, with a return to the baseline in 3–4 h after intake [32]. However, several differences in their pharmacological profiles were observed. Particularly, NEP demonstrated a faster onset and earlier disappearance of subjective effects. In contrast, NEH displayed weaker peak effects, with a delayed onset and a shorter duration. The effects of both substances generally returned to baseline within 3–4 h post-administration. These short-duration effects are consistent with the pharmacological profiles of several SCs [32,33]. Moreover, the amphetamine-like molecular scaffold indicates central nervous system-stimulating and sympathomimetic effects similar to those of amphetamine and its derivatives. The observed pharmacological profiles are similar to those of mephedrone and cathinone, characterized by the euphorigenic and stimulant properties [27,30]. The early oral fluid peak and rapid onset are typical of SCs, independently of the route of administration. Indeed, these observations were also reported for the oral consumption of mephedrone and methylone [25,27,30], 3-MMC [34] and MDMA [25,27,35,36,37,38,39,40,41]. Furthermore, the T-C NEH and NEP effects are similar to those of self-administered cocaine and/or amphetamine or methamphetamine [42,43,44,45,46]. Notably, the effect results were considered averages across the considered dose range, avoiding any correlations to a specific dose.

To date, there has only been a limited number of documented clinical cases of NEH and NEP intoxications, these mostly involving polydrug consumption-related intoxication or driving under the influence. In these cases, plasma and/or urine were the selected matrices for toxicological quantitative analyses [16,17,24]. Oral fluid NEH and NEP analytical detection was conducted only in a retrospective study of NPS prevalence among psychiatric patients or patients in addiction care clinics; in this study, qualitative analyses were performed [47]. As a result, the elimination half-life of NEH or NEP was not determined. Otherwise, the plasma elimination half-life for MDMA, methylone and mephedrone ranges between 10 and 12 h, 5 and 6 h, and 2 and 3 h, respectively [25,29].

Finally, the study presents some limitations strictly related to the naturalistic–observational design, such as the different settings, expectancies and the lack of a placebo control. Although randomized, double-blind, placebo-controlled studies are the gold standard with which to evaluate the subjective effects of substances, previous drug consumption can affect the validity of blinding/a placebo control due to the expectations of effects based on previous experiences. In two previously published double-blind and placebo-controlled clinical trials of mephedrone, MDMA and methylone were evaluated in participants with previous experience in the consumption of designer drugs (such as MDMA) and other psychostimulants [25,30]. After the administration of mephedrone, MDMA or methylone, between 83% and 94% of subjects correctly identified the consumed designer drug/psychostimulant. Furthermore, between 92% and 94% of subjects correctly identified that they received a placebo.

As expected in our study, all the participants identified the substance they self-administered as a designer drug/psychostimulant, based on the felt effects. Notably, the results of the subjective/physiological effects observed in other naturalistic–observational studies were very similar to those observed in double-blind placebo-controlled studies carried out by our group and others. This similarity in the profile of pharmacological effects has been described in studies administering MDMA [25,28,30,48,49,50], mephedrone [25,26,27,51], methylone [28,29,30], 2C-B [52,53] or 5-MeO-DMT [53,54]. The main effects were very similar in both designs, although some differences were found in the intensity and magnitude of the subjective feelings when taking into account the different doses studied. The mentioned results reinforce the validity, in some cases, of the observational approach and the results presented in the present paper. Indeed, the best approach to studying the subjective effects of drugs remains a double-blind, placebo-controlled study design, though this depends on whether this is permitted with regards to the ethics.

Another limitation of this study is the small sample size, and the limited genders, potentially limiting the detection of meaningful dose- or gender-specific effects. Furthermore, the limited number of subjects likely impacted the results, particularly when using Dunnett’s post hoc test. The test may not have had enough statistical power to detect true differences with fewer subjects, potentially leading to the overlooking of significant effects that might have been identified in a larger sample. In addition, the small sample size limited the formal testing of normality assumptions.

On the other hand, the following strengths should be noted: the participation of female subjects, the dose selection by the participants according to their preferences, the inclusion of two different substances, the inclusion of subjects who were already experienced with the same or a similar NPS, the recreational scenario, and the use of a validated methodology used in controlled studies (rating scales, questionnaires) and analytic techniques.

Furthermore, given the challenges associated with plasma and blood sampling in both clinical and naturalistic settings, such as invasiveness, logistical complexity, and participant compliance, the findings underscore the value of oral fluid as an alternative biological matrix. The use of oral fluid allows for non-invasive, rapid, and repeated sample collection, which is particularly advantageous in studies involving psychoactive substances administered in real-world scenarios. As such, this approach may facilitate the expansion of pharmacokinetic and toxicological research on NPS, including NEH and NEP, and may contribute to improved monitoring in both clinical and forensic settings.

## 4. Materials and Methods

The design of this study was a naturalistic, prospective, observational study, with minimal intervention in recreation drug users who self-administered one dose of intranasal NEH or intranasal NEP. The methodology, including procedures and evaluations, coincides with previous observational–naturalistic studies aimed at evaluating the acute effects of other NPS [26,27,28]. We included 16 subjects (9 men and 7 women). Participants brought their own dose obtained from an unknown source, which was tested by Energy Control, a harm reduction organization that provides a drug checking service to drug users (AsociaciónBienestar y Desarrollo, https://energycontrol.org/). NEH and NEP were analyzed by gas chromatography coupled to mass spectrometry (GC-MS), through a large panel method allowing the detection of the most frequent drugs of abuse, such as MDMA, cocaine, heroin, amphetamine and methamphetamine, LSD, and multiple NPS (methylone, mephedrone, and other synthetic cathinones, synthetic cannabinoids, tryptamines, among others). The test showed more than 95% purity for NEH and NEP, as well as the absence of toxic components or adulterants [26,27,28].

Participants were recreative drug users who had experience with amphetamines, ecstasy and/or synthetic cathinones at least once in their lifetimes without experiencing previous serious adverse reactions. Exclusion criteria included a history of any serious medical or psychopathological disorder including substance use disorder (except for nicotine), a previous serious adverse reaction in users of amphetamines, ecstasy (MDMA) and synthetic cathinones, and use of chronic medication. Participants were recruited by word-of-mouth through the harm reduction, non-governmental organization Energy Control (AsociaciónBienestar y Desarrollo-ABD). The study protocol was approved by the Clinical Research Ethics Committee of the Hospital Universitari Germans TriasiPujol (CEIC HUGTiP, Badalona, Spain; PI-18-267). The study was conducted in accordance with the Declaration of Helsinki recommendations and Spanish biomedical regulations (Biomedical Research Law 14/2007).

All the participants were fully informed, both orally and in writing, about the study characteristics. All of them indicated their agreement to participate and signed an informed consent prior inclusion. Subjects were financially compensated for their participation.

The dose of self-administered intranasal NEP and intranasal NEH was selected by the participants, based, presumably, on their previous experience. Eight subjects self-administered NEP intranasally, and the mean dose was 33.75 mg (men 37.50 mg; women 30 mg; range 20–40 mg) (3 men insufflated 40 mg and 1 man 30 mg; 1 woman 40 mg, 2 women 30 mg, and 1 woman 20 mg). Eight subjects self-administered NEH intranasally, and the mean dose was 32.50 mg (3 men insufflated 40 mg and 2 men 30 mg; 2 women 30 mg and 1 woman 20 mg). All the selected doses were well tolerated, and no serious adverse events were observed. No local tissue damage to the nostrils or any other potential acute medical complication after snorting was reported.

### 4.1. Procedures

Sessions were conducted in a private club closed to the public for the study, to which participants were summoned at 15:00 h and where they stayed until the end of the session at 20:00 h. Upon arrival, urine samples were collected to screen for the presence of conventional drugs (amphetamine, barbiturates, benzodiazepines, cocaine, ecstasy, methadone, methamphetamine, morphine/opiate, TCA, and cannabinoids) using the Nal von Minden Drug-Screen^®^ Multi TD Urine test. A pregnancy test was performed in women using the Clio Test Plus^®^.

Subjects were not allowed to use any recreational drug one week prior to the session or consume alcohol or caffeinated beverages in the previous 24 h. Participants received instructions and training on the procedures and questionnaires used throughout the sessions. The sessions were conducted in a naturalistic setting. Participants were allowed to talk, read, listen to music, or play games, except during the evaluation times. However, they were asked to refrain from talking about the effects of the substance.

All the subjects were evaluated at baseline (pre-dose), at 20 min (0.33 h) and 40 min (0.66 h), and at 1, 1.5, 2, 3 and 4 h after self-administration of NEP or NEH, which occurred approximately at 16:00 h. At each time point, evaluations were followed in a specific order: physiological effects, oral fluid collection, and subjective effect scales and questionnaires.

### 4.2. Physiological Effects

Non-invasive SBP and DBP, and HR, were determined with an Omron^®^ monitor (Omron, Hoofddorp, The Netherlands), at baseline, 20 min, 40 min, and 1, 1.5, 2, 3 and 4 h after administration. Cutaneous temperature was measured at the same times (baseline, 20 and 40 min, and 1, 1.5, 2, 3 and 4 h after administration).

### 4.3. Subjective Effects

Subjective effects were measured using a set of VAS items, the 49-item ARCI and the VESSPA.

The VAS items, for 100 mm, ranging from “not at all” to “extremely”, were used to rate some items such as “intensity”, “stimulated”, ” high”, “good effects”, “bad effects”, “liking”, “changes in distances”, “changes in colors”, “changes in shapes”, “changes in lights”, “hallucinations (seeing lights or spots)”, “changes in hearing”, hallucinations (hearing sounds or voices)”, “drowsiness”, “concentration”, “dizziness”, “confusion”, “different or changed body feeling”, “unreal body feeling”, “different surroundings”, “unreal surroundings”, “open”, “trust”, “feeling close to others”, “I want to be with other people”, “I want to hug someone”, “sexual desire”, and “sexual arousal”.

The Spanish validated version of the short-form ARCI is a true/false 49-item questionnaire, an instrument for the determination of subjective drug effects [30]. It includes five subscales related to the following: drug sedation (pentobarbital-chlorpromazine-alcohol group, PCAG), euphoria (morphine-benzedrine group, MBG), dysphoria and somatic symptoms (lysergic acid diethylamide group, LSD), intellectual efficiency and energy (benzedrine group, BG) and d-amphetamine-like effects (amphetamine, A).

The VESSPA questionnaire is a questionnaire that enables the measurement of changes in subjective effects caused by different drugs, including stimulants and psychedelics. It includes six subscales: sedation (S), psychosomatic anxiety (ANX), changes in perception (CP), pleasure and sociability (SOC), activity and energy (ACT), and psychotic symptoms (PS) [30].

The VAS questionnaire administered at baseline, 0.33 h (20 min) and 0.66 h (40 min), and at 1, 1.5, 2, 3 and 4 h after drug administration, with the exception of “sexual desire” VAS and “sexual arousal” VAS items, and the ARCI and VESSPA forms were completed at baseline, and 1, 2, 3 and 4 h after self-administration of the drug.

### 4.4. Oral Fluid Concentrations and Statistical Analysis

Oral fluid samples were collected with Salivette^®^ tubes at baseline, at 20 and 40 min and at 1, 1.5, 2, 3, and 4 h after self-administration. Samples were then centrifuged and frozen at −20 °C until analysis. The detection and quantification of NEH and NEP in oral fluid concentrations were analyzed using a previously validated GC-MS/MS method by our research group [55].

For physiological variables (SBP, DBP, HR, and T) and subjective (VAS, ARCI, and VESSPA) variables, we calculated differences with respect to the baseline. Peak effects (E_max_) were determined and the area under the curve of the effects (AUC_0−4h_) was calculated using the trapezoidal rule by the Pharmacokinetic Functions for Microsoft Excel (Joel Usansky, Atul Desai, and Diane Tang-Liu, Department of Pharmacokinetics and Drug Metabolism, Allergan, Irvine, CA, USA).

Firstly, a two-way analysis of variance (ANOVA) was conducted to evaluate the influence of dose and gender on the calculated parameters. As the results showed only marginally statistically significant interactions between dose and gender, and for each factor individually, the analysis was deemed inconclusive. Consequently, the subsequent statistical analysis was performed without considering these factors, grouping doses and genders into a single group for NEP and for NEH.

Dunnett’s multiple comparison post hoc test was conducted to evaluate the effects along time for each drug, NEH and NEP, comparing the different time points with the baseline (times 0–20 min, 0–40 min, 0–1 h, 0–1.5 h, 0–2 h, 0–3 h and 0–4 h). Student’s t-test for unpaired samples was performed to compare the E_max_ and AUC_0−4h_ values of all parameters calculated between NEH and NEP. Differences in time to reach peak effects (T_max_) between NEH and NEP were assessed using a non-parametric test (Wilcoxon test).

Additionally, a GLM ANOVA with drug (NEH and NEP) and time (baseline, 20 min, 40 min and 1, 1.5, 2, 3 and 4 h) as factors was used to compare the time course (T-C) of effects between NEH and NEP,

For NEH and NEP oral fluid concentrations, the maximum concentration (C_max_), the time needed to reach the maximum concentration (T_max_) and the AUC_0−4h_ were calculated using the Pharmacokinetic Functions for Microsoft Excel (Joel Usansky, Atul Desai, and Diane Tang-Liu, Department of Pharmacokinetics and Drug Metabolism, Allergan, Irvine, CA, USA).

Statistically analyses were performed using PAWS Statistics version 18 (SPSS Inc., Chicago, IL, USA). Statistical significance was defined as *p* < 0.05.

## 5. Conclusions

The results presented constitute the outcome of a preliminary approach to the acute physiological and subjective effects of NEH and NEP. These findings suggest that intranasal self-administration of NEH and NEP in experienced recreational drug users, within a non-controlled setting, induces a constellation of psychostimulant-like effects. Oral fluid concentration analysis may present a suitable alternative to plasma or urine for the quantification of NEH and NEP in clinical and toxicological research, offering a simple, non-invasive method for sample collection. Thus, it may represent a critical advancement for future pharmacological, toxicological, and forensic investigations involving NPS. However, further controlled experimental studies under controlled conditions are required to substantiate our observational data and to compare the pharmacological profiles of NEH and NEP in humans with those of other NPS and traditional pharmacological agents and classical drugs of abuse, as well as to validate and standardize this methodology.

Thus, the incorporation of alternative biological matrices such as oral fluid may represent a critical advancement for future pharmacological, toxicological, and forensic investigations involving NPS, as well as for validating and standardizing this methodology.

## Figures and Tables

**Table 1:** Statistical results for physiological effects observed after intranasal administration of NEH (*n* = 8) and NEP (*n* = 8).

	NEH (*n* = 8)			NEP (*n* = 8)		*t*-Student		ANOVA		
Variable	Parameter	Mean ± SD	Dunnett’s Test	Mean ± SD	Dunnett’s Test	T	*p*-Value	F	*p*-Value	T-C
SBP	E_max_	22.13 ± 12.56		20.63 ± 10.63		0.258	0.800			
	AUC_0–4_	41.02 ± 33.55		44.68 ± 26.03		−0.244	0.811			
	T-C		**a**, **b**, **c**, **d**, e		**a**, **b**, **c**, **d**, **e**			0.325	0.941	--
DBP	E_max_	16.75 ± 9.50		11.88 ± 10.30		0.985	0.341			
	AUC_0–4_	34.73 ± 33.94		23.48 ± 23.74		0.769	0.455			
	T-C		**a**, **b**, c, d, e		**a**, b, d, g			4.590	**<0.001**	h
HR	E_max_	21.63 ± 10.05		22.31 ± 11.11		−0.130	0.899			
	AUC_0–4_	39.60 ± 30.41		46.21 ± 37.67		−0.386	0.705			
	T-C		**a**, **f**		**a**, **b**, c, d, e, **f**, **g**			0.253	0.970	--

Abbreviations: NEH, *N*-ethylhexedrone; NEP, *N*-ethylpentylone; SBP, systolic blood pressure measured by mmHg; DBP, diastolic blood measured by mmHg; HR, heart rate, measured by beats per minute; T, cutaneous temperature measured by °C; E_max_, peak effects 0–4 h (differences from baseline); AUC_0–4_, area under the curve between 0 and 4 h; T-C, time course. For Dunnett’s test, statistical differences are presented as “**a**” *p* < 0.01 (times 0–20 min), “b” *p* < 0.05, “**b**” *p* < 0.01 (times 0–40 min), “c” *p* < 0.05, “**c**” *p* < 0.01 (times 0–1 h), “d” *p* < 0.05, “**d**” *p* < 0.01 (times 0–1.5 h), “e” *p* < 0.05, “**e**” *p* < 0.01 (times 0–2 h) and “**f**” *p* < 0.01 (times 0–3 h), “g” *p* < 0.05, “**g**” *p* < 0.01 (times 0–4 h). For ANOVA, statistical differences between for NEH and NEP are presented as “h” *p* < 0.05.

**Table 2:** Summary of the results regarding the subjective effects observed after the self-administration of NEH (*n* = 8) and NEP (*n* = 8).

	NEH			NEP		*t*-Student		ANOVA		
Variable	Parameter	Mean ± SD	Dunnett’s Test	Mean ± SD	Dunnett’s Test	T	*p*-Value	F	*p*-Value	T-C
Intensity	E_max_	32.88 ± 20.50		55.50 ± 19.57		−2.258	**0.040**			
	AUC_0–4_	67.50 ± 55.71		108.55 ± 81.58		−1.176	0.259			
	T-C		**a**, **b**, **c**, **d**, **e**		**a**, **b**, **c**, **d**, e			2.055	0.056	--
Stimulated	E_max_	37.13 ± 22.39		59.50 ± 23.69		−1.941	0.073			
	AUC_0–4_	78.17 ± 58.51		115.16 ± 94.36		−0.650	0.526			
	T-C		**a**, **b**, **c**, **d**, **e**		**a**, **b**, **c**, **d**, **e**, f					
High	E_max_	34.63 ± 21.60		51.13 ± 17.25		−1.688	0.113			
	AUC_0–4_	66.93 ± 56.96		70.65 ± 30.84		−0.162	0.874			
	T-C		**a**, **b**, **c**, **d**, **e**		**a**, **b**, **c**, **d**			2.893	**0.009**	NS
Good effects	E_max_	36.38 ± 23.23		61.00 ± 14.85		−2.526	**0.024**			
	AUC_0–4_	71.03 ± 60.00		85.40 ± 22.49		−0.706	0.492			
	T-C		**a, b, c, d,** e		**a, b, c, d**			3.226	**0.004**	c
Bad effects	E_max_	3.88 ± 4.83		40.13 ± 36.88		−2.756	**0.015**			
	AUC_0–4_	4.06 ± 5.06		71.29 ± 101.56		−1.870	0.083			
	T-C		NS		c, d			2.289	**0.033**	d
Liking	E_max_	38.63 ± 21.76		72.38 ± 16.95		−3.461	**0.004**			
	AUC_0–4_	71.81 ± 50.34		94.28 ± 21.78		−1.159	0.266			
	T-C		**a**, **b**, **c**, **d**, **e**		**a**, **b**, **c**, **d**			7.074	**<0.001**	**a**, **b**
Drowsiness	E_max_	4.00 ± 7.87		29.00 ± 34.62		−1.992	0.066			
	AUC_0–4_	4.26 ± 8.37		52.53 ± 88.29		−1.540	0.146			
	T-C		NS		e			2.250	**0.036**	NS
Concentration	E_max_	31.63 ± 25.48		48.63 ± 23.10		−1.398	0.184			
	AUC_0–4_	64.08 ± 56.58		73.99 ± 31.01		−0.434	0.671			
	T-C		**a**, **b**, **c**, **d**, **e**		**a, b,** c, d			1.953	0.069	--
Dizziness	E_max_	2.88 ± 5.00		5.50 ± 14.35		−0.489	0.633			
	AUC_0–4_	3.28 ± 5.15		12.14 ± 32.85		−0.753	0.464			
	T-C		NS		NS			0.887	0.520	--
Different body feeling	E_max_	27.88 ± 24.63		34.25 ± 21.71		−0.549	0.592			
	AUC_0–4_	54.69 ± 51.36		63.00 ± 55.44		0.715	0.486			
	T-C		**c**, **e**		**c**			1.138	0.348	--
Open	E_max_	40.00 ± 26.55		65.88 ± 18.43		−2.264	**0.040**			
	AUC_0–4_	76.75 ± 63.71		114.01 ± 46.17		−1.340	0.202			
	T-C		**a**, **b**, **c**, **d**, **e**		**a**, **b**, **c**, **d**, f			2.314	**0.032**	h
Trust	E_max_	38.75 ± 28.74		64.38 ± 20.04		−2.069	0.058			
	AUC_0–4_	75.54 ± 63.94		126.47 ± 57.88		−1.671	0.117			
	T-C		**a**, **b**, **c**, **d**, **e**		**a**, **b**, **c**, **d**, **e**, f			2.042	0.057	--
Feeling close to others	E_max_	42.75 ± 28.08		65.25 ± 23.49		−1.738	0.104			
	AUC_0–4_	82.22 ± 64.49		119.35 ± 59.51		−1.187	0.255			
	T-C		**a**, **b**, **c**, **d**, **e**		**a**, **b**, **c**, **d**, f			1.822	0.091	--
I want to be with other people	E_max_	27.75 ± 30.21		67.50 ± 34.98		−2.432	**0.029**			
	AUC_0–4_	58.64 ± 70.59		122.95 ± 75.39		−1.761	0.100			
	T-C		**a**, **b**, **c**, **d**, **e**		NS			3.026	**0.006**	b, c
I want to hug someone	E_max_	27.25 ± 29.47		34.38 ± 28.73		−0.490	0.632			
	AUC_0–4_	55.27 ± 66.29		78.64 ± 77.79		−0.647	0.528			
	T-C		**b**, **c**, **d**		**a**, **b**, **c**, **d**, e			0.887	0.520	**--**
ARCI-PCAG	E_max_	−2.25 ± 1.16		4.25 ± 4.06		−4.351	**0.001**			
	AUC_0–4_	−4.31 ± 3.92		5.88 ± 9.26		−2.864	**0.012**			
	T-C		c		**--**			2.814	**0.034**	f, g
ARCI-MBG	E_max_	4.88 ± 3.94		10.75 ± 9.47		−0.204	0.841			
	AUC_0–4_	10.88 ± 10.88		1.50 ± 4.00		0.025	0.981			
	T-C (df=)		**c**, **e**		**c**, e			1.002	0.414	--
ARCI-LSD	E_max_	0.88 ± 2.10		1.75 ± 3.01		−0.6.74	0.511			
	AUC_0–4_	1.13 ± 4.32		4.56 ± 7.47		−1.127	0.279			
	T-C		--		c			1.053	0.388	--
ARCI-BG	E_max_	3.50 ± 2.33		1.75 ± 4.17		1.037	0.317			
	AUC_0–4_	7.88 ± 5.93		4.06 ± 9.71		0.948	0.359			
	T-C		**c**, **e**,f		--			1.218	0.313	--
ARCI-A	E_max_	4.13 ± 2.85		4.88 ± 2.75		−0.536	0.601			
	AUC_0–4_	10.56 ± 7.48		11.00 ± 7.60		−0.116	0.909			
	T-C		**c**, **e**, **f**		**c**, **e**, f, **g**			0.980	0.426	--
VESSPA-S	E_max_	0.34 ± 0.40		1.04 ± 0.93		−1.964	0.070			
	AUC_0–4_	0.44 ± 0.50		2.31 ± 2.53		−2.049	0.060			
	T-C		NS		**e**, f, g			2.237	0.077	
VESSPA-ANX	E_max_	1.46 ± 1.14		1.67 ± 0.93		−0.398	0.697			
	AUC_0–4_	3.46 ± 3.09		4.29 ± 3.42		−0.511	0.617			
	T-C		**c**, **e**, f		**c**, **e**, **f**, **g**			0.572	0.684	--
VESSPA-CP	E_max_	0.00 ± 0.00		0.06 ± 0.12		−1.433	0.174			
	AUC_0–4_	0.00 ± 0.00		0.06 ± 0.12		−1.433	0.174			
	T-C		NS		NS			2.053	0.099	--
VESSPA-SOC	E_max_	1.31 ± 1.11		1.42 ± 0.87		−0.213	0.834			
	AUC_0–4_	2.54 ± 2.55		2.60 ± 2.03		−0.055	0.957			
	T-C		**c**		c, g			1.394	0.248	--
VESSPA-ACT	E_max_	1.79 ± 0.99		2.04 ± 0.73		−0.578	0.573			
	AUC_0–4_	3.75 ± 2.20		3.79 ± 1.45		−0.044	0.965			
	T-C		**c**, **e**		c, e			0.978	0.427	--
VESSPA-PS	E_max_	0.29 ± 0.35		0.63 ± 0.71		−1.185	0.256			
	AUC_0–4_	0.58 ± 0.65		1.42 ± 1.69		−1.294	0.217			
	T-C		**c**		NS			0.636	0.639	

Abbreviations: NEH, *N*-ethylhexedrone; NEP, *N*-ethylpentylone; SBP, E_max_, peak effects 0–4 h (differences from baseline); AUC_0–4_, area under the curve between 0 and 4 h; T-C, time course; NS, non-significant. For Dunnett’s test, statistical differences are presented as “**a**” *p* < 0.01 (times 0–20 min), “**b**” *p* < 0.01 (times 0–40 min), “**c**” *p* < 0.01 (times 0–1 h), “d” *p* < 0.05, “**d**” *p* < 0.01 (times 0–1.5 h), “e” *p* < 0.05, “**e**” *p* < 0.01 (times 0–2 h) and “f” *p* < 0.05, “**f**” *p* < 0.01 (times 0–3 h), “g” *p* < 0.05, “**g**” *p* < 0.01 (times 0–4 h). For ANOVA, statistical differences between for NEH and NEP are presented as “**a**” *p* < 0.01 (time 0 h), “b” *p* < 0.05, “**b**” *p* < 0.01 (time 20 min), “c” *p* < 0.05, “d” *p* < 0.05, “f” *p* < 0.05, “g” *p* < 0.05,and “h” *p* < 0.05.

## Data Availability

The original contributions presented in the study are included in the article, further inquiries can be directed to the corresponding author.
